# From Implementation Towards Change Management: A Plea for a Multi-stakeholder View on Innovation Implementation: Comment on "What Managers Find Important for Implementation of Innovations in the Healthcare Sector – Practice Through Six Management Perspectives"

**DOI:** 10.34172/ijhpm.2022.7202

**Published:** 2022-07-09

**Authors:** Monika Martens, Hilario Lorenzo Xin Chen, Josefien van Olmen

**Affiliations:** ^1^Department of Public Health, Institute of Tropical Medicine, Antwerp, Belgium.; ^2^Department of Family Medicine and Population Health (FAMPOP), Faculty of Medicine and Health Sciences, University of Antwerp, Antwerp, Belgium.; ^3^Former McKinsey & Company, Amsterdam, The Netherlands.

**Keywords:** Change Management, Innovation, Implementation, Organisational Change, Healthcare Organisation, Theoretical Management Perspectives

## Abstract

In their 2021 paper, Palm and Persson Fischier focus on the enabling factors that can facilitate innovation implementation, specifically the step of moving from idea generation to implementation in a healthcare context. The authors address the lack of concretisation of theoretical perspectives related to the implementation of innovations and hence propose to work holistically with six management perspectives. Our commentary provides new interdisciplinary angles to the six perspectives, from management and organisation literature to theory of change management. This provides future innovation managers with different viewpoints and inspires creative thinking and reflection. Our commentary also critiques the emphasis on the enablers and hence a constructionist-based approach to change management. We plea that a focus on the ‘good, bad, and ugly’—or rather all moods of change—is warranted in order to support holistic and successful change.

## Introduction

 Innovations in healthcare can be drivers of change that have the potential to improve health outcomes and patient experiences. Introducing innovations, although necessary, is a difficult endeavour as it involves the typical challenges of the implementation phase that are related to changing mind-sets and behaviours. Seminal work in management literature on change management teaches us that 70% of all change programmes fail.^1,2^ Newer studies point to the same amount of failed implementations of such programmes.^1,3^

 In their 2021 paper, Palm and Persson Fischier^4^ focus on the enabling factors that can facilitate innovation implementation, specifically the step of moving from idea generation to implementation in a healthcare context. This approach simplifies implementation into a two-step process, rather than distilling implementation into different phases from idea generation to piloting, scale up, and embedding within routines, norms and beliefs of the organisation/health system, ie, institutionalisation. Their study brings various new and useful insights about managing innovation in a healthcare context to the forefront (summarised in Table 2 in their paper, which contains 35 enabling factors).^4^ Another strength of this study is the participatory action research design, which allowed healthcare practitioners to engage with and concretise theoretical management perspectives with practical content for successful implementation of innovations. Within this design, the authors made a reasoned choice for tools and theories such as Design Thinking, Dialogic Organisational Development theory, and back casting to engage participants.

 Apart from the study’s strengths, we found two important limitations. A first pitfall is that the snowballing (or pearl growing) technique they used failed to identify a few relevant papers in the domain of (change) management and organisation, which potentially could have been addressed by identifying a more comprehensive start set. Badampudi and colleagues^5^ indicate: “*snowballing can potentially be more reliable than a database search however, the reliability is highly dependent on the creation of a suitable start set.”* While two theories from the field of organisation and management were identified through the snowballing technique, we believe including more literature from the field of general organisational theory, and specifically, theory of change management could have been useful. For example, ‘change management’ could have been included as a keyword in their literature search or consulting a management scientist could have provided guidance into some of the seminal works in organisational change. A second limitation in our opinion is that their description of innovation (page 2) as *“exploring new solutions *[exploration]* in contrast to incremental development exploiting existing solutions *[exploitation]*”* is still rather vague and simplistic and does not take into account complexity principles and characteristics, eg, of emergence (emergent behaviours), path dependence, multiple feedback loops, and unintended consequences. An important criticism as put forward by Lehoux and colleagues^6^ and van Olmen and colleagues^7^ is that not all innovations are intrinsically good. In contrast, Palm and Persson Fischier^4^ argue that innovations should not be normative but rather drive increased value in healthcare. While this is meant to be the aim of innovations, their impact and consequences (positive, negative, and unintended) remain unknown before implementation. There may be winners and losers. Rather than assuming innovations add value per se, an innovation can be regarded as an injection of resources and opportunities into a health system.^7^ For a successful innovation, this system, therefore, needs to have the capacity to transform these into desired outputs—the ‘absorption capacity.’^8^ This definition of an innovation as an input into a complex system may prove more useful. Moreover, the authors’ conceptualisation of innovation as inherently good ignores different perspectives (actors, values, and/or ideations) and also has implications on their approach to solely focus on the enablers of innovation implementation. We argue that such an emphasis is one-sighted (or one-sided) and neglects negative aspects and consequences.

 We elaborate on these limitations and call for (1) an interdisciplinary approach drawing upon key literature from the disciplines of management, health, psychology, and complexity thinking; and (2) a multi-stakeholder perspective on the ‘good, bad, and ugly’ of change management.

## The Added Value of Theoretical Models of Change Management

 The six management perspectives proposed by the authors^4^ are useful yet limited. They could have drawn more from various disciplines, and specifically from management and organisation literature and theory of change management, which remained an untapped source of information. This would have provided a stronger methodological and theoretical basis. We, therefore, wish to highlight several additional insights from management literature on organisational change (Table 1). We emphasise that this list is not comprehensive and that we focussed on theories that we deemed to have a practical value for healthcare managers.

**Table 1:** Additional Change Management Frameworks and Theories

**Model/Frame**	**Description**	**Source**
The five minds of a manager	1) Managing self: the reflective mind-set 2) Managing organisations: the analytic mind-set 3) Managing context: the worldly mind-set 4) Managing relationships: the collaborative mind-set 5) Managing change: the action mind-set	Gosling and Mintzberg^9^
Influence model	Incorporates four building blocks of change: 1) Role modelling 2) Fostering understanding and conviction 3) Capacity building by developing talent and skills 4) Reinforcing with formal mechanisms Points 1 and 2 propose leadership approaches, 3 and 4 organisation approaches. Points 1 and 3 offer an indirect approach to change implementation while 2 and 4 are direct approaches to support change by creating awareness and belief about the need to change and incentives. Keller and Aiken^1^ add nine inconvenient truths about these four building blocks: 1) Role Modelling 1. Your leaders believe they already are the change 2. Influence leaders are not that influential 2) Understanding and Conviction 3. What motivates you doesn’t motivate your employees (there are at least five sources of meaning and motivation: having an impact on society, beneficiary/customer, organisation, working team, and self; cfr. five minds of a manager) 4. You’re better off letting them write their own story (listening not telling creates ownership) 5. It takes both + and − to create real energy 3) Capability Building 6. Employees are what they think 7. Good intentions are not enough: time, space, and energy is required to do something additional, or even to do something in a new way 4) Reinforcing Mechanisms 8. Money is the most expensive way to motivate people (small, unexpected rewards have disproportionate effects on employees’ motivation during change programs) 9. A fair process is as important as a fair outcome	Keller and Aiken^1^
Configurations of organisational structure	Mintzberg suggests a typology of five basic organisational configurations: Simple Structure, Machine Bureaucracy, Professional Bureaucracy, Divisionalized Form, and Adhocracy. Furthermore, he distinguishes (*a*) five coordinating mechanisms and (*b*) five key parts of the organisation. Each of the five configurations relies on one of the five coordinating mechanisms and tends to favour one of the five parts: 1) Simple Structure has (*a*) direct supervision as prime coordinating mechanism; and (*b*) strategic apex as key part in the organisation. 2) Machine Bureaucracy has (*a*) standardisation of work processes as prime coordinating mechanism; and (*b*) technostructure as key part in the organisation. 3) Professional Bureaucracy has (*a*) standardisation of skills as prime coordinating mechanism; and (*b*) operating core as key part in the organisation. 4) Divisionalized Form has (*a*) standardisation of outputs as prime coordinating mechanism; and (*b*) the middle line as key part in the organisation. 5) Adhocracy has (*a*) mutual adjustment as prime coordinating mechanism; and (*b*) support staff as key part in the organisation.	Mintzberg^10^
Multipolar performance assessment framework	This framework integrates four key organisational functions (goal attainment, service production, adaptation to the environment, and values and culture) and the tensions between these functions (which call for contextual, strategic, tactical, operational, allocation and legitimisation alignment).	Sicotte et al^11^
Eight-step change model	Kotter describes eight steps of implementing change powerfully and successfully: 1) Establishing a sense of urgency 2) Creating the guiding coalition 3) Developing a change vision 4) Communicating the vision for buy-in 5) Empowering broad-based action 6) Generating short-term wins 7) Consolidate improvements 8) Incorporating into the culture	Kotter^2^

 The frameworks presented in Table 1 have less of a mechanistic, constructionist-based managerial view and relate more to leadership and balancing effects. For example, Gosling and Mintzberg’s^9^ framework on the five mind-sets of managers recognises the role of mind-sets in change management and how these interact with behaviours. As the authors argue, managers have to fulfil a multitude of often contrary expectations and there is an overemphasis on what they have to accomplish vs. on how they have to think.^9^ Yet,* “everything that every effective manager does is sandwiched between action on the ground and reflection in the abstract. (…) Every manager has to (…) function at the point where reflective thinking meets practical doing.”*^9^ Similar to the six managerial perspectives described by Palm and Persson Fischier,^4^ these authors put forward five sets of the managerial mind; five ways in which managers interpret and deal with the world around them: the reflective, the collaborative, analytic, worldly, and action mind-set. According to the authors, *“change, to be successful, cannot follow some mechanistic schedule of steps, of formulation followed by implementation. Action and reflection have to blend in a natural flow.”*^9^ That has to include collaboration, minding the organisation, and wider context. It follows that the action mind-set pulls everything together through the process of change—within the self, relationships, the organisation, and context. While some managers are more reflective, more analytical, or action-oriented, there is a need to weave all of these mind-sets together.

 Another model useful in deriving actions-to-change—from idea generation to implementation—is the influence model,^1^ described in Table 1. Psychology research, suggests that four basic conditions are necessary before employees will change their behaviour^1^: (*a*) a compelling story, that creates understanding and conviction, because employees must see the point of the change and agree with it; (*b*) role modelling, because they must also see leadership and colleagues they admire behaving in the new way; (*c*) reinforcing mechanisms, because systems, processes, and incentives must be in line with the new behaviour; and (*d*) capability building, because employees must have the skills required to make the desired changes. While these conditions are rational and common sense, Keller and Aiken^1^ argue that managers also need to deal with the irrational (and often unconscious) nature of how humans interpret their environment and how they choose to act, and the unintended consequences thereof. Given the difficulty of change and beneficiaries’ resistance and uncertainties, serious time and energy investment is needed; the failure to formalise and create the space for practice back in the workplace dooms most change programs.^1^

 Furthermore, a link can be made to Mintzberg’s configurations of organisational structure.^10^ Most healthcare organisations are mechanistic and/or professional bureaucracies, where standardisation of work processes and skills, respectively, are the main coordinating mechanism. Bureaucracies are typically risk-averse, siloed, procedural, hierarchical, and efficient but slow to respond to both internal change and external forces and shocks.^12^ Agile organisations—adhocracies in Mintzberg’s^10^ terminology—in contrast, are collaborative, responsive, and action-oriented.^12^ These types of structures impact care processes and health outcomes. For example, providing integrated healthcare services requires professional collaboration across specialisations, levels, and sectors, which provides challenges in many siloed organisations and bureaucratic systems. Aside from processes and outcomes, structures also impact how change is perceived and therefore implemented. In line with the organisational goals and structure, if efficiency is the main operational target (with no room for experimentation), then it will be more difficult to allow for the appropriate time and training for change management programmes and empathetic engagement in healthcare systems.^13^

Table 2 depicts how these additional frameworks presented in Table 1 are related to the six theoretical management perspectives by Palm and Persson Fischier^4^ and also draw from various disciplines. Practical lessons and potential methods concretising frameworks into action are drawn up.

**Table 2:** The Six Theoretical Management Perspectives and Additional Theoretical Insights From Change Management, Complexity Thinking, and Organisation Structure Theory

**Six Theoretical Management Perspectives**	**Additional Theoretical Insights From Change Management, Complexity Thinking and Organisation Structure Theory **	**Practical Lessons and Potential Methods Derived From These Additional Theoretical Insights With Concrete Examples**
(*a*) Collaboration with the beneficiaries (ie, patients and other healthcare stakeholders) for the healthcare effort	Link to fostering understanding and conviction, as a building block in the influence model^1^; dealing with the ‘moods’ of organisational change, also uncertainties and resistance^14^ and link to the collaborative mind-set^9^	Moving beyond ‘people as resources or assets,’ managing ‘relations,’ adopting a less controlling and more engaging managing style, eg, regular two-way feedback, listening and reacting actively to create ownership, and facing resistance by being explicit about the + and − of change, eg, when dealing with digital health technologies (administrative burden and time away from patient contact vs. reducing inefficiencies and increasing quality of care, whilst delivering population health management).
(*b*) Collaborations with other relevant stakeholders in the implementation process	Link to the collaborative mind-set^9^ and strategic alignment in multipolar performance assessment framework^11^	Managing and engaging in external ‘relations,’ eg, by setting up a network with actors of common interests; anticipating interests via a network analysis; SWOT; or eg, a stakeholder analysis as a strategy for identifying and selecting stakeholders for policy dialogue about scale-up of integrated care for chronic diseases.
(*c*) Organisational culture	Organisational culture can be strongly linked to organisation structure^10^; link to maintaining values and culture in multipolar performance assessment framework^11^ and link to the worldly/context mind-set,^9^ ie, about getting into other people’s circumstances, habits, cultures	Realising organisational culture may be one of the most difficult things to change, because of its link to organisational structure and its embeddedness in societal values and complexities. Organisational cultural change can be induced from the outside world, eg, the increased use of teleconsultations due to the COVID-19 pandemic; or can also come from within the organisation, often individuals, eg, inspirational leaders, managers or local champions, who may illustrate change ad-hoc and bottom-up: eg, showing respectful attitude towards patients and junior staff members in a strongly hierarchical healthcare environment. The creation of an ombudsman and of options for intervision procedures can create safe environments for such change agents.
(*d*) Human resource management	Link to capacity building, as a building block of change in the influence model^1^	Organising training and offering education, eg, within the implementation of the WHO’s PEN interventions in Cambodia, healthcare staff was trained in screening and follow-up care for diabetes and hypertension.
(*e*) Organisational structure	Link to organisational structure theory: mechanistic/professional bureaucracy vs. adhocracy,^10^ link to the analytical/organisational mind-set^9^	Reflecting on impact of organisational structure on processes and outcomes and mind-sets/perceptions about change, by means of Mintzberg’s organisational configurations and its characteristics (pitfalls and benefits), eg, start-ups set up their structure from scratch and typically choose for a flat organisation, befitting of the collaborative, entrepreneurial and creative mind-set they want to induce. Hospitals in contrast, as (historically) often public institutions act more as mechanistic/professional bureaucracies, visualised in their specialised departments. For structural reform, they may practically need a new spacial design to foster multidisciplinary collaboration, to install a space where different specialisations are assembled.
(*f*) Resource availability	Link to formal mechanisms, as a building block of change in the influence model^1^ and allocation alignment in multipolar performance assessment framework^11^	Offering small rewards and searching for the right incentives, eg, results-based financing.
(*g*) A seventh perspective in the table is described, yet not labelled: *“the importance of being clear about why the organization needs to implement a new solution, but does not directly provide any input to answer the question of how the innovation should be implemented”*^4^	Link to fostering understanding and conviction, as a building block of change in the influence model^1^ and goal attainment in multipolar performance assessment framework^11^	A compelling story; this requires extensive dialogue to generate wide support, buy-in and a shared vision about the ‘why’ behind any change, eg, why digital healthcare technologies are not just about monitoring and evaluation, but also improve quality of care for patients and help physicians to streamline processes and reduce the burden of (user-unfriendly) administration. Co-creation of such a tool with a focus group of respected physicians and healthcare staff can make up a compelling story.
Additional psychological perspectives warrant: Dealing with mind-sets, moods and behaviours	Various behaviour change theories… Link to the reflective mind-set^9^ and the ‘moods’ of organisational change^14^	Overall, a stronger focus should be given on ‘how to’ change mind-sets, moods and behaviours, whereas these are the hardest to influence and are the main reasons why most change programmes fail.^1,2^ For example, a capacity-building programme for healthcare managers may improve their motivation, and therefore, their intention toward positive organisational change, instilling a ‘can-do’ attitude, or in other words, a desire for change, whilst putting knowledge and skills to effective use.
Additional complexity perspectives warrant: Dealing with complex systems	Multipolar performance assessment framework^11^	A focus on the coherence between values and goals of an organisation and how they interact with the external context. Also attention for the fact that organisational performance is much influenced by internal social interactions and relations. The awareness of both issues can help managers to make sense of unintended effects of financial incentives (eg, pay for performance) on communication and collaboration within healthcare teams.

Abbreviations: WHO, World Health Organization; PEN, Package of Essential Non-communicable Diseases Interventions; COVID-19, coronavirus disease 2019; SWOT, Strengths, Weaknesses, Opportunities, and Threats.

## Enablers of Innovation Implementation vs. the Moods of Change

 While we encourage increased attention on the enablers instead of solely barriers to implementation, it is highly doubtful that a manager—or any healthcare practitioner—does not have to address barriers to innovation implementation and specifically deal with resistance to change. There are different reasons why change is difficult: individual factors (including habits, security and stability, economic and emotional factors, such as fear of the unknown) and organisational factors (including structural and/or group inertia, limited focus of change, threat to expertise or established power). Palm and Persson Fischier^4^ argue for an emphasis on the enablers and hence a constructionist-based approach to change management. Their study uses methodologies of Dialogic Organisational Development and back casting to focus on visions for the future rather than problems. The authors claim that *“a more traditional emphasis on ‘problems’ may make stakeholders focus on different aspects, which hinders, rather than enables, innovation.”*^4^ While it is true that a focus on ‘what’s wrong’ may induce change fatigue, at the same time, to foster understanding and conviction, a mix of deficit-based and constructionist-based approaches is needed.^1^ An important inconvenient truth as presented in the influence model states that it takes both positive and negative to create real energy.^1^

 More so, we should be aware that organisational change is linked to different moods which impact organisational momentum and productivity: (1) denial (and uncertainty), (2) resistance (and fear), (3) exploration (withdrawal or testing and acceptance), and (4) commitment (or commitment to the adaptation of the innovation).^14^ Little empirical research has been conducted on the emotional impact of organisational change, but these ‘different moods of organisational change’ provide leeway to frame different attitudes towards change and develop suitable strategies to deal with them. In management literature, ‘organisational health’ is commonly used and measured to understand how to start the implementation of change programmes, whereas some will require a lot more work to change the mind and behaviours of staff members than others.^15^ Resistance to change is widely described yet causal explanations underlying resistance and contingency strategies on dealing with the ‘ugly’ side (negative and irrational behaviours and emotions) less so. Therefore, we would argue that a focus on the ‘good, bad and ugly’—or rather all moods of change—is warranted to support holistic and successful change.

## Conclusion

 We laud Palm and Persson Fischier’s methodological approach to participatory action research whilst engaging various healthcare practitioners, but see further opportunities for more rigorous systematic and/or interdisciplinary literature review into other theoretical perspectives on the innovation process from idea generation to implementation. We have added theoretical insights to the six management perspectives which can be tested, evaluated, and conceptualised further in order to generate an even more comprehensive and practical frame that is relevant to the healthcare context and keeps into account the need for a combination of both deficit-based and constructionist-based approaches to change management.

 A limitation to our commentary is that our review was also not extensive; rather, we focussed primarily on management literature (specifically, models summarizing practical steps) to further draw from that field and its untapped potential for learning that can be transferred and applied to the healthcare field. While practical applications of change management may vary contextually for well-resourced vs. under-resourced health systems, we believe that these models are relevant across contexts (though different perspectives and mind-sets may be prioritised) and in dealing with change in crises situations such as the coronavirus disease 2019 (COVID-19) pandemic (when action took priority over reflection). We emphasise that strengthening change management and the innovation capacity of health systems can enhance the resilience of health systems dealing with unforeseen sudden changes.

 While the authors^4^ focus on the enablers of innovation implementation, we argue that simultaneously closer attention needs to be paid to the reason why 70% of all change programmes fail, namely how to deal with the real challenge in the implementation phase: mind-sets, behaviours, and moods (including resistance to change). Various authors indeed point out that failure to change is typically linked to people’s mind-sets and behaviours.^1,2^ Additionally we would like to stress the need for empathetic engagement with beneficiaries and stakeholders in change programmes. Hence, we call for further research on behaviours, mind-sets, and feelings of stakeholders in innovation implementation, including managers, healthcare workers, other implementers, (internal/external) support staff for coaching, education and administration, and beneficiaries or users of the innovation, namely the patients and/or health practitioners themselves.

## Acknowledgements

 We would like to sincerely thank Ritwik Dahake for his collaboration and meticulous work on editing, proofreading and enhancing the manuscript.

## Ethical issues

 Not applicable.

## Competing interests

 Authors declare that they have no competing interests.

## Authors’ contributions

 MM, HLXN, and JvO conceptualised, designed, and drafted this commentary. All authors have read the manuscript and approved it for submission.
